# Maternal Mortality Ratio and Causes of Death in IRI Between 2009 and 2012 

**Published:** 2016-09

**Authors:** Marzieh Vahiddastjerdy, Nasrin Changizi, Abas Habibollahi, Leila Janani, Zahra Farahani, Farah Babaee

**Affiliations:** 1 Department of Obstetrics and Gynecology, Arash Hospital, Tehran University of Medical Sciences, Tehran, Iran; 2 Maternal, Fetal and Neonatal Research Center, Tehran University of Medical Sciences, Tehran, Iran; 3 Department of Epidemiology and Biostatistics, School of Public Health, Tehran University of Medical Sciences, Tehran, Iran; 4 Ministry of Health and Medical Education, Tehran, Iran

**Keywords:** Maternal Mortality, Epidemiology, Etiology, Hospitals, Iran

## Abstract

**Objective:** The Maternal Mortality Ratio is an important health indicator. We presented the distribution and causes of maternal mortality in Islamic Republic of Iran.

**Materials and methods:** After provision of an electronic Registry system for date entry, a descriptive-retrospective data collection had been performed for all maternal Deaths in March 2009- March 2012. All maternal deaths and their demographic characteristic were identified by using medical registries, death certificates, and relevant codes according to International Classification of Diseases (ICD-9) during pregnancy, labor, and 42 days after parturition.

**Results:** During 3 years, there were 5094317 deliveries and 941 maternal deaths (MMR of 18.5 per 1000000 live births). We had access to pertained data of 896 cases (95.2%) for review in our study. Of 896 reported deaths, 549 were classified as direct, 302 as indirect and 45 as unknown**.** Hemorrhage was the most common cause of maternal mortality, followed by Preeclampsia, Eclampsia and sepsis**.** Among all indirect causes, cardio -vascular diseases were responsible for 10% of maternal deaths, followed by thromboembolism, HTN and renal diseases.

**Conclusion:** Although maternal mortality ratio in IRI could be comparable with the developed countries but its pattern is following developing countries and with this study we had provided reliable data for other prospective studies.

## Introduction

Maternal mortality ratio (MMR) as a health system indicator refers to the number of pregnancy related maternal death in 100000 live births (1, 2).

The estimated number of maternal mortality worldwide in 2008 and 2013 were reported about 358000 and 293,000 by WHO (3, 4). Different countries have different portions of this global rate from 5-10/100000 in developed countries to 85-1570/100000 in Oceania and Sub-Saharan African countries. Statistics shows that 99% of maternal deaths occur in developing countries (5, 6).The Highest Ratios of maternal deaths in 2008 belong to India (63000) and lowest ratio was seen in Greece (2/100000) (1). 

Causes of Maternal death are categorized in to 2 groups; direct and indirect. Direct causes include pregnancy-related deaths occurring during pregnancy period up to 42 days Postpartum. Indirect causes refer to pre-existing or new medical-psychological problems which develop by pregnancy such as heart disease. Some maternal deaths are also categorized as coincidental death which occur during pregnancy or the puerperium but are not related to pregnancy, like car accident (7, 5). Based on WHO report About 80% of maternal deaths in Africa are related to five direct causes including; hemorrhage, sepsis, pregnancy-induced hypertension, rupture of uterus and complications of abortion (8). Post partum hemorrhage and infection are main causes of maternal mortality in developing countries while in developed countries, pulmonary embolus and stroke are seen more commonly (1). 

Some delays were mentioned as predisposing factors in maternal death including delay in seeking required medical help, delay in seeking a timely medical facility and delay in giving enough care(Delay in Managing ) (9). 

Lots of efforts have been applied to achieve the fifth millennium Development Goal (MDG5) for reduction MMR by 75% from 1995 to 2105 (9); Maternal education, public health care accessibility, training of staffs and patients, access to prenatal and postnatal care, earlier diagnosis, treatment of medical and obstetric problems, lifesaving procedures and interventions to promote efficient care and then reducing maternal mortality (10). Economical aspects also have effects in this reduction (11). Moreover universal access to reproductive health care and some local strategies like treatment of HIV/Aids have had played an appreciable impact on regional and global MMR (12).

In Asia, Japan with 7 (per 100000) maternal mortality rate in 2008 had the lowest ratio, followed by South Korea and Taiwan (11 and 14) (5). Based on WHO Report, Maternal mortality ratio of The Islamic Republic of Iran has declined from 150/100000 in 1990 to 19.5/100000 in 2012. Iran like Sri Lanka and Malaysia is one of few countries which had achieved MDG5 (13, 1). Regarding paperbased National Maternal mortality Surveillance system in Iran, it seems that there are more rooms for reducing maternal deaths so we decided to assess the maternal mortality ratio and explore main causes of maternal deaths during 3 years between March 2009 and March 2012 by designing an electronic base for Maternal Mortality Surveillance system with more detailed items. Our results included main causes of maternal death and their leading factors that can provide comparable data source to other publications.

## Materials and methods

A descriptive-retrospective data collection has been performed from March 2009 till March 2012.Inclusion criteria were all maternal mortality reported between 2009-2012/based on ICD9 definition of maternal death. We considered all maternal deaths during pregnancy, labor and 42 days after parturition. Based on National Maternal Mortality Surveillance System (NMMSS), (A system Installed in IRI from 2001, paper records that include all the Maternal Mortalities records based on ICD-9 Definition). After preparing an electronic software for Data Entry with more detailed items in comparison to national NMMSS, we get through all Recorded Maternal Mortality Cases in this System in more detailed approaches than NMMSS (their demographic characteristic data such as nationality, age, parity, socioeconomic status, educational level, occupation, reproductive and medical/obstetric history, mode of delivery, history of them through hospitalization and referral problems including Errors in management ). Based on the definition, causes of maternal death were also categorized in 2 direct and indirect groups according to International Classification of Diseases. Direct causes consisted of hemorrhage (Bleeding > 500 ml), sepsis, preeclampsia, eclampsia, ectopic pregnancy, abortion-related problems, molar pregnancy, Amniotic fluid embolism, fatty liver and anesthesia related complications. Indirect causes composed of cardiovascular disease, diabetes mellitus, hypertensive disorders, HIV syndrome, peritonitis, renal and GI diseases. We also divided hemorrhagic causes in 3sub groups; bleeding before and during and or after delivery. 

This 3 year retrospective survey was done with getting help of 50 technical officers acquainted with NMMSS, who were trained in filling the Software designed in InfoPath especially for this project. These technicians after confirming all the data recorded in each dead mother’s file and with supervision of 50 Obstetricians in this regard, filled the Software based questionnaire .For each maternal Death regarding its main cause, Quantitative variables were reported as Mean (SD), while qualitative variables were reported through Frequencies (Percentages).

Ethics approval for the study was obtained from the institutional review board of Ministry of Health and Medical Education (ID; 90-04-159-17419). All data were considered secret.

## Results

During 3 years study period, there were 5094317 deliveries and 941 maternal deaths were reported (MMR of 18.5 per 1000000 live births). We had access to pertained data of 896 cases (95.2%) for review in our study. Mean Maternal age was 29.84 ± 6.64 years; 9.5 % were < 20 years and 11.8% > 40 years, mean gestational age at the time of death was reported 32.4 ± 8.83 weeks, 28.7% were primigravida, 19.7% grand multigravida (gravida > 6), 3.3% were migrant (Afghan and other), 156 (18.5%) of cases were illiterate, 88.9% were housekeeper and 95.2% of maternal deaths occurred in low-income families. Detailed demographic data is shown in table 1. 

**Table 1:** Characteristics of participants

**Number of Participants**	**n = ** **896**
Age (years)	Mean ± SD 29.84 ± 6.64
Gestational Age (years)	Mean ± SD 32.04 ± 8.83
Education	Number (%)
Illiterate (%)	156 (18.5 %)
Lower diploma	605(71.6%)
University (%)	84 (9.9%)
No response (%)	51 (-)
Job	n (%)
House Wife	779 (88.9%)
Worker	75 (8.6%)
Student	9 (1.0%)
Other	13 (1.5%)
No response	20 (-)
Nationality	n (%)
Iranian (%)	863 (96.7%)
Afghan (%)	24 (2.7%)
Other (%)	5 (0.6 %)
No response (%)	4 (-)

Of 896 reported deaths, 549 were classified as direct, 302 as indirect and 45 as unknown maternal deaths. Data showed that Hemorrhage was the most common cause of maternal mortality (31 cases before delivery and 184 cases, during and post partum), followed by Preeclampsia, Eclampsia and sepsis whereas HIV and Molar pregnancy were the least important cause (0 and 0.1%). Relatively few cases died due to Anesthesia related complication (1.3%). Among all indirect causes, cardio vascular disease was responsible for 10% of maternal deaths, followed by thromboembolism, HTN and renal disease. Table 2 demonstrates the clinical cause of death. 

**Table 2:** Causes of maternal deaths in Iranian Hospitals between 2009 and 2012

**Cause of Death**	**Frequency**	**%**
Direct		
Bleeding before delivery	6	0.7%
Bleeding during delivery	25	3.0%
Bleeding after delivery	184	20.5%
Before delivery sepsis	20	2.2%
After delivery sepsis	31	3.5%
Amniotic fluid Emboli	29	3.2%
Regional anesthesia complication	3	0.3%
General anesthesia complication	9	1.0%
Fatty liver	13	1.4%
Preeclampsia	69	7.7%
Eclampsia	53	5.9%
Abortion	30	3.3%
Ectopic Pregnancy	10	1.1%
Molar pregnancy	1	0.1%
Other	66	7.4%
All direct	549	61.3%
Indirect		
Cardiovascular	90	10.0%
Human Immunodeficiency Virus	0	0.0%
Diabetes Mellitus	2	0.2%
Errors	4	0.4%
Bowel perforation	6	0.7%
Renal diseases	15	1.7%
Peritonitis	4	0.4%
Tuberculosis	8	0.8%
Chronic Hypertension	17	1.8%
Trumbo embolism	33	4.0%
Other	123	13.7%
All indirect	302	33.7%
Unknown	45	5.0%
Total	896	100

Most of maternal deaths (664) occurred after termination of pregnancy; of which 60.9% of patients had cesarean delivery and 1.1% had instrumental delivery. Three Cases with pharmacologic delivery also died because of direct and indirect causes; maternal deaths in relation to mode of delivery are shown in Table 3.

**Table 3:** Maternal deaths in relation to mode of delivery

**Type of childbirth**	**Direct**		**Indirect**		**Unknown**		**Total death**	
	**n**	**Percent**	**n**	**Percent**	**n**	**Percent**	**n**	**Percent**
NVD*	168	39.5%	67	35.1%	8	28.6%	243	37.7%
C/S**	252	59.2%	122	63.9%	19	67.9%	393	60.9%
Forceps	1	0.2%	0	0.0%	0	0.0%	1	0.2%
Vacuum	4	0.9%	0	0.0%	0	0.0%	4	0.9%
Pharmacologic	1	0.2%	2	1.0%	1	3.5%	4	0.6%
No response	12	-	7	-	0	-	19	-
Total	438	100.0%	198	100.0%	28	100.0%	664	100.0%

* Normal vaginal delivery;

** Cesarean section

Maternal Mortality cases had been mostly among those gravida 1 (28/7%) and Gravida 2-3(42/6%) (Table 4).

The final destination of most of Maternal Mortalities had been public Educational (54.8%) and Public Non-Educational (28/7%) Hospitals (Table 5).

Maternal Mortalities mostly occurred in poor families (Table 6).

More than Half of Maternal Death Final Destination and place of death had been level 3 of Hospitals (Hospitals equipped with NICU, ICU and different Subspecialties) (Table 7).

93.3% of Maternal Death occurred in Hospitals (Table 8).

In 41.3% of Maternal Deaths are some sorts of errors with major Substandard Care (Table 9).

There had been some sort of delay in 61.9 % of cases mostly Delay in Hospital Management (34.9%) and Delay in Decision Making (27.7%) (Table10).

**Table 4:** Gravida and Maternal Mortality

**Gravid Group**	**Direct**		**Indirect**		**Unknown**		**Total death**	
	**n**	**Percent**	**n**	**Percent**	**n**	**Percent**	**n**	**Percent**
1	143	26.0%	96	31.8%	18	40.0%	257	28.7%
2-3	226	41.2%	140	46.4%	16	35.6%	382	42.6%
4-5	108	19.7%	49	16.2%	8	17.8%	165	18.4%
>= 6	72	13.1%	17	5.6%	3	6.6%	92	10.3%
Total	549	100.0%	302	100.0%	45	100.0%	896	100.0%

**Table 5:** Type of Hospitals and Maternal Mortality

**Type of hospital**	**Direct**		**Indirect**		**Unknown**		**Total death**	
	**n**	**Percent**	**n**	**Percent**	**n**	**Percent**	**n**	**Percent**
Public Educational	253	53.8%	145	54.9%	22	66.7%	420	54.8%
Public Noneducational	137	29.1%	80	30.3%	3	9.1%	220	28.7%
Public other organs	12	2.6%	6	2.3%	1	3.0%	19	2.5%
Private	30	6.4%	14	5.3%	3	9.1%	47	6.1%
Charity	4	0.9%	3	1.1%	1	3.0%	8	1.0%
Social Security	33	7.0%	16	6.1%	3	9.1%	52	6.8%
Azad University	1	0.2%	0	0.0%	0	0.0%	1	0.1%
No response	79	-	38	-	12	-	129	-
Total	549	100.0%	302	100.0%	45	100.0%	896	100.0%

**Table 6:** Level of Monthly Income and Maternal Mortality

**Level of Income**	**Direct**		**Indirect**		**Unknown**		**Total death**	
	**n**	**Percent**	**n**	**Percent**	**n**	**Percent**	**n**	**Percent**
< 329$	493	94.6%	279	95.9%	37	97.4%	809	95.2%
330-699$	21	4.0%	10	3.4%	0	0.0%	31	3.6%
700$-1000$	7	1.4%	2	0.7%	1	2.6%	10	1.2%
No response	28	-	11	-	7	-	46	-
Total	549	100.0%	302	100.0%	45	100.0%	896	100.0%

## Discussion

There are many publications that provide valuable statistic data of maternal mortality in Iran, (14, 15) but our study is the first nationally representative study of detailed maternal mortality risk factors in Iran. 

Based on our results the maternal mortality ratio (observed in approximately 5,094,317 Live births) was 18.5 per 100,000 over the 3 years study period. While based on WHO reports in 2008 and 2010, this ratio had been 30 and 19.5/100000 respectively and the difference is because of WHO estimates of Missing Data (1, 13). The estimated MMR per 100,000 live births is 920 in Africa, 330 in Asia, and 10 in Europe (8). 

**Graph 1:**
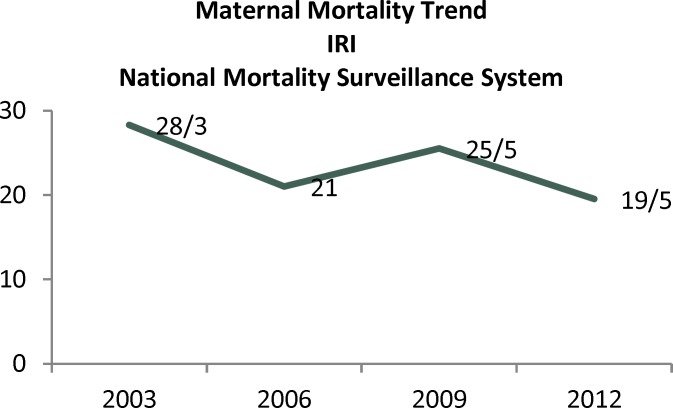
Trend of MMR in IRI was shown based on National Maternal Mortality Surveillance System (Ministry of Health & Medical Education Website)

Our results showed that Iran is one of the few Asian countries which had achieved MDG5.Luckily over the last decade, registration of maternal deaths in Iran also demonstrates a dramatic decrease (Graph1). In modern Obstetrics, accessibility of 24-hour blood bank services, Routine antenatal care, recognition and timely management of medical illnesses, universal access to reproductive health care, antibiotic therapy, provision of risk management guide lines, employment of skilled birth attendance are the main reasons of this decreasing pattern (16, 1). 

Unlike Developed Countries, the direct causes of maternal mortality contributed much significantly (61%) than indirect causes (36.7%) in Maternal Mortality. This reveals that direct causes are still the major factors in maternal deaths in our country where as in developed countries indirect causes of death are dominant. According to another report from Iran (Yazd province) the ratio of direct to indirect cause was 3:1 (14). Our finding was comparable with other developing countries; In Nigeria74.75% and in India 81.8% of maternal mortality was reported due to direct causes (17, 18) while Denmark with MMR 9.8 per 100,000 during the period 1985–1994 had 29 direct and 31 indirect maternal deaths.

According to this study, we found that the leading cause of maternal death was Hemorrhage, with most deaths occurring in Intra-partum or Post-partum period. Karimzadeh et al. also reported that hemorrhage was the commonest cause of death in their province (Yazd-Iran) which was followed by eclampsia, infection and embolism, however; Moazzeni has pointed to pulmonary embolism, diabetes mellitus and pregnancy related hypertensive disorders as the commonest cause of Iranian mothers' death (1, 14, 19).

**Table 7:** Hospital Level Of Maternal Death

**Level of hospital**	**Direct**		**Indirect**		**Unknown**		**Total death**	
	**n**	**Percent**	**n**	**Percent**	**n**	**Percent**	**n**	**Percent**
Level1	34	8.2%	21	9.7%	2	6.4%	57	8.6%
Level2	141	33.8%	72	33.4%	6	19.4%	219	33.0%
Level3	242	5.8%	123	56.9%	23	74.2%	388	58.4%
No response	132	-	86	-	14	-	232	-
Total	549	100.0%	302	100.0%	45	100.0%		100.0%

**Table 8:** Place of Maternal Death

**Place of delivery**	**Direct**		**Indirect**		**Unknown**		**Total death**	
	**n**	**Percent**	**n**	**Percent**	**n**	**Percent**	**n**	**Percent**
Hospital	388	91.9%	184	96.3%	26	92.9%	598	93.3%
Home	24	5.7%	3	1.6%	1	3.6%	28	4.4%
Childbirth facility	7	1.7%	4	2.1%	0	0%	11	1.7%
In the way	2	0.5%	0	0%	1	3.6%	3	0.5%
During Transport	1	0.2%	0	0%	0	0%	1	0.2%
No response	16	-	7	-	0	-	23	-
Total	438	100%	198	100%	28	100%	664	100%

**Table 9:** Type of Management

**Type of Management **	**Direct**		**Indirect**		**Unknown**		**Total death**	
	**n**	**Percent**	**n**	**Percent**	**n**	**Percent**	**n**	**Percent**
Appropriate	92	20.8%	77	32.6%	12	50.0%	181	25.7%
Inappropriate with minor substandard care	131	29.6%	91	38.6%	10	41.7%	232	33.0%
Inappropriate with major substandard care	220	49.6%	68	28.8%	2	8.3%	290	41.3%
No response	106	-	66	-	21	-	193	-
Total	549	100.0%	302	100.0%	45	100.0%	896	100.0%

It is reported that every year about 14 million pregnant women experience postpartum hemorrhagic complications due to retained placenta, lower genitalia lacerations, uterine atony and rupture. Prata et al. believed that by postpartum hemorrhage management especially in low-resource settings 55- 82% of MMR could be reduced (20). Postpartum hemorrhage was also named as the commonest cause of maternal death in an Indian tertiary center (52.5 %) (7). Provision of standardized guidelines for Prevention and management of Obstetrical Hemorrhage, Including implementation of effective and appropriate treatments, and well-identified risk factors are considered beneficial Solutions (21, 22).

Our analysis showed that most deaths occurred in postpartum period that may be related to etiological differences. This finding was compatible with other reports revealing postpartum hemorrhage as the main cause of death (18). About 18 % of Yemeni maternal death happens in prenatal period and 82 % during parturition and afterwards. Post partum hemorrhage that is responsible for a quarter of mother’s death is preventable with some interventions like using uterotonic agents, manual expulsion of placenta, blood transfusion and timely hysterectomy (22, 23). But the most important point is Risk assessment before conception and through pregnancy, for better management of mothers in an appropriate center with adequate expertise and facilities. The second most frequent complication leading to maternal death was hypertensive disorder (13.6%). Our findings are the same as other Iranian research (3, 15) but the rate is higher than other Asian countries. Hypertensive disorders contributed to 50.000 maternal deaths annually in the world, Khan et al. estimated the proportion of maternal deaths due to hypertensive disorders to be 9% in South Asian countries and Montgomery et al. reported 7% in India (18, 24, 25). Our results showed that the MMR due to hypertension was still very high in spite of availability of magnesium sulfate and well trained professionals in the managing pre-eclampsia in our centers). 

**Table 10:** Type of Delay

**Type of Delay**	**Direct (549)**	**Indirect (302)**	**Unknown (45)**	**Total death (896)**
Delay	368(67.0%)	165(54.6%)	22(48.9%)	555(61.9%)
Delay in decision making	166(30.2%)	74(24.5%)	8(17.8%)	248(27.7%)
Delay in referral	99(18.0%)	50(16.6%)	4(8.9%)	153(17.1%)
Delay in hospital management	218(39.7%)	87(28.8%)	8(17.8%)	313(34.9%)
Errors and neglects	135(24.6%)	64(21.2%)	6(13.3%)	205(22.9%)

In spite of widespread use of antibiotics during pregnancy and delivery, Sepsis had a great role in maternal death in our country (5.7%).

Although deaths due to abortion are not high in our country, it seems most of them could be preventable. Abortion-related complications are sepsis, hemorrhage, uterine perforation and lower genital tract injuries (26).

Among all indirect causes, cardiovascular disease was responsible for 10% of maternal death. Eftekhar et.al also showed cardiac disease accounting for 9.4% of maternal mortality in their publication (15).

We also found that maternal death was more common in mothers who underwent cesarean section than vaginal delivery. Based On recent report by WHO the rate of Cesarean in IRI was 48% in one year (27). Butin Maternal Death cases it had been almost 61% which implies emergency cases in most of them (64.9%) in which General Anesthesia had been performed (69%). We guess that all medical, obstetrical, surgical and anesthetic factors may influence on the maternal death, however; mortality probability for all emergency caesarean sections are six times more than vaginal delivery (28). Several publications reported a dramatic increase in risk of maternal mortality with increasing rate of cesarean delivery (29). In another report between 122 Asian countries China, Sri Lanka, Vietnam, and Thailand with higher rates of caesarean section than Cambodia, India, Japan, 

Nepal, and the Philippines, Operative vaginal delivery and caesarean section are associated with increased risk of maternal mortality rate independently (30).

Based On other study from Iran, 95% of childbirths took place in hospitals and the rest of births were homebirths or on the way to hospital (31). Approximately the same percentage of maternal deaths was observed in our study. 

Finally we found that illiteracy, Young and old age pregnancy, primi and grand multi parity, low socioeconomic status, living in rural area, are contributing factors in increasing possibility of maternal deaths as other studies have shown (6, 9). Clark et al. cited and confirmed that about 28-40% of maternal deaths could be preventable (32). Main et al. believed that maternal mortality should not be considered a single clinical criteria and deceasing this rate needs in-depth evaluation of individual causes of death (33). However Sajedinejad et al. showed that decreasing maternal mortality requires dealing with various factors other than individual causes of death. All political will, reallocation of national resources (especially health resources) in the governmental sector, education, attention to the expansion of the private sector trade and improving spectrums of governance should be involved (34).

There is no doubt that focusing on the cause of death improves pregnancy outcome and can reduce maternal mortality ratio. We believe this study has several strengths. Lots of efforts have been applied to achieve the fifth millennium Development Goal (MDG5), but there is no much time until 2015 and any attributable investigation would be helpful. We also presented the cause of death datasets with their descriptive details. Our study is the first nationally representative study of maternal mortality in Iran that provide comparable data source to other publications. Although there are certain limitations to this study, our analysis study covered only 3 years data. In addition accidental or incidental maternal deaths were not considered.

## Conclusion

Although maternal mortality ratio in Iran could be comparable to developed countries, its pattern is still consistent with developing countries and since based on this study, we have detailed information about what had been going on in each part of IRI about Maternal deaths, we suggest in depth study of maternal mortalities with special attention to those cases with major substandard care, and to those parts of country with higher deaths terminated with Cesarean section.

Also we suggest using the software provided in this project for gathering more detailed information In National Maternal Surveillance System in IRI.
